# Effect of Simulated Mechanical Filtration by the Stylus Tip on Changes in the Parameters of Areal Surface Texture

**DOI:** 10.3390/ma18092060

**Published:** 2025-04-30

**Authors:** Rafal Reizer, Andrzej Dzierwa, Wieslaw Żelasko, Zuzanna Pawlus

**Affiliations:** 1 Institute of Materials Engineering, Faculty of Exact and Technical Sciences, University of Rzeszow, Pigonia Street 1, 35-310 Rzeszow, Poland; 2 Faculty of Mechanical Engineering and Aeronautics, Rzeszow University of Technology, Powstancow Warszawy 8 Street, 35-959 Rzeszow, Poland; adzierwa@prz.edu.pl; 3 Faculty of Mechanics and Technology, Rzeszow University of Technology, Kwiatkowskiego Street 4, 37-450 Stalowa Wola, Poland; w.zelasko@prz.edu.pl; 4 Faculty of Medical Sciences in Katowice, Medical University of Silesia, Poniatowskiego Street 15, 40-055 Katowice, Poland; s78730@365.sum.edu.pl

**Keywords:** stylus tip, surface texture, measurement, parameters

## Abstract

The objective of this work was to study distortion of the 3D surface textures measurement using the stylus tip technique. This distortion was simulated using a dilation procedure. Topographies of many machined surfaces of various characters measured using a white light interferometer were analysed. The growth in the tip of the radius of the stylus produced larger changes in surface texture. Mechanical filtration by a stylus tip caused a reduction in the amplitude parameters, hybrid parameters, and average radius of the peak curvature. Variations in skewness (Ssk) and kurtosis (Sku) depended on the type of surface. The depth and density of the furrows had a tendency to decrease. The measurement results were more distorted for a higher roughness amplitude and a smaller main wavelength.

## 1. Introduction

The surface topography consists of the error of form, waviness, and roughness. The waviness and roughness together are called surface texture. Surface texture affects the functional properties of machine elements such as contact, sealing, adhesion, friction, and wear [1,2]. It is important in other scientific disciplines, such as medicine, including dermatology [3,4,5]. Therefore, the accurate measurement of surface texture is very important. Unfortunately, the measurement of surface topography is sensitive to errors [6]. These errors depend on the measurement method. The stylus tip technique is the most typical method of 2D profile measurement. This method used a stylus tip that motion is changed into a signal versus horizontal position [7,8]. The tip of the stylus is typically made of diamond or sapphire. These materials are chosen for their hardness, durability, and ability to accurately trace the surface profile without causing damage. Recently, the measurement of areal (3D) surface topography became popular and provides accurate information because the analysis of 2D profiles can lead to false results [9]. The first areal measurements were performed using the stylus technique. An additional dimension was required in the measurement system [10,11]. Recently, the measurement of areal surface topography became more common using optical methods [12,13], mainly due to the much shorter measurement time than the application of the stylus tip profilometer. Optical methods are sensitive to measurement errors [14], caused by various sources, such as the occurrences of non-measurement points [15,16], spikes [17], high-frequency noise [18,19], and surface stitching [20]. However, for some types of surfaces, such as those with hard light-reflecting characteristics, resulting in a high number of non-measured points, the application of a stylus tip device is necessary. Because the measurement of the stylus tip is well known, it can be used as a reference method for surface topography measurement. It is also used in fields other than mechanical engineering. For example, the measurement of skin surface replicas was performed using the stylus tip technique [21,22,23,24,25].

Errors in surface topography measurement using stylus devices are related to tip velocity [1]. At high speeds, it is difficult to achieve contact between the surface and the tip of the stylus because of the stylus flight. The possibility of stylus flight depends on the construction of the pick-up, speed, force, and type of surface texture. The impact of the stylus velocity was modelled in [26,27,28]. Arvinth Davinci [29], Pawlus and Smieszek [30], and Pawlus et al. [31] analysed changes in areal (3D) surface topography parameters, caused by an increase in the speed of the stylus tip.

The measurement of areal surface topography using a stylus tip device is time-consuming. Changes in temperature affected the values of surface texture parameters [32,33].

**Figure 1:**
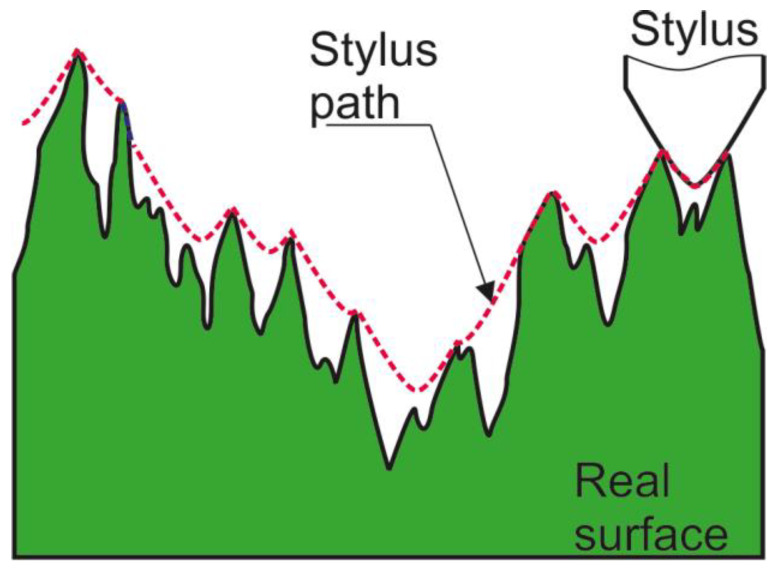
Mechanical filtration of the stylus tip, in accordance with [6,34].

The most important problem is related to the effect of the size of the stylus tip on the results of areal surface texture measurement. This effect is called mechanical filtration, because it is similar to low-pass filtering. The finer surface details (low wavelengths) are eliminated. This behaviour depends mainly on the radius of the stylus tip; in addition, the impact of the cone angle is smaller. The stylus typically has a problem reaching the bottom of the narrow valleys (Figure 1).

The smallest measured wavelength is close to the size of the stylus tip [35]. The tip radius of 2 µm causes the low distortion of the profiles of machined surfaces [1,2]. However, a stylus tip radii of 5 and 10 µm can also be used. Substantial distortions of profiles can be found after the application of stylus tip radii of 5 [36] and 10 µm [37]. The distortion of two-process surfaces (having traces of two processes) is more complicated than that of one-process surfaces (having traces of one process) [38]. The styli are often flattened during wear, and their radii increase. Typical profile distortions were analysed, and an amplitude decrease due to the increase in the stylus tip radius was found. Zahouani et al. [39] studied the effect of the radius of the tip of the stylus on various areal parameters of surface texture measurement. Lee [40] and Lee and Cho [41] proposed the radius of the tip of the stylus based on surface characteristics consisting of the minimum wavelength and rms. height.

The radius of the tip of the stylus is related to the minimum sampling interval, which should be higher than the size of the tip radius [1,42]. The selection of the sampling interval depends on the purpose of the investigations. Pawlus and Chetwynd [43] selected a sampling interval to recognise honing valleys. Rosen and Garnier [44] chosen the sampling interval taking into account changes in surface height. Thomas and Rosen [45] and Pawlus and Zelasko [46] considered rough contact mechanics.

It is evident from the review of the literature that mainly the distortion of 2D profiles due to mechanical filtration by a stylus tip was analysed; only in a few works were distortions of 3D surface topography studied. In most cases, only changes in surface amplitudes due to changes in the tip radii were analysed. This work tries to fill this gap. By simulation, distortions of various machined surface textures due to increasing the stylus tip radius were studied, and the effects of tip radius on various parameters (amplitude, spatial, hybrid, and feature) were analysed.

## 2. Materials and Methods

The topographies of many (45) machined surfaces were measured using the Talysurf CCI Lite white light interferometer, produced by Taylor Hobson (Leicester, UK). It applies an innovative correlation algorithm to find the coherence peak and phase position of an interference pattern produced by the precision optical scanning unit. Surfaces with reflectivity between 0.3% and 100% can be measured using a single algorithm. A variety of objectives are available. All types of materials are measurable. The vertical resolution is 0.01 nm and vertical range is 2.2 mm.

Surfaces were created using various processes such as milling, polishing, lapping, grinding, one-process honing, plateau honing, abrasive blasting, abrasive blasting followed by lapping, laser texturing, and texturing using burnishing. Surface textures were isotropic, anisotropic, or mixed, after one or two processes (plateau honing, abrasive blasting followed by lapping and texturing using laser and burnishing). Rectangular samples made of grey cast iron after honing and texturing using burnishing (20 mm length, 12 mm width, and 4 mm thickness) were cut from cylinder liners of 130 mm diameter. The other circular steel samples had diameter of 25 mm and thickness of 7.9 mm. This work was focused on analysing the behaviour of very different surface types. The aim was to analyse surface topographies with a high range of amplitudes. Therefore, surface assessed by rms. height (Sq) was between 0.01 and 6 µm. Table 1 presents information on the measured samples.

In measurements, using a Talysurf CCI Lite white light interferometer, a 5× objective lens was applied. The measuring area of 3.29 mm × 3.29 mm contained 1024 × 1024 data points. The measured area is the consequence of used objective. Three measurements were taken for each individual sample. After measurements, flat surface topographies were levelled, whereas for curved surfaces, forms were removed by the polynomial of the second level. Then, the non-measured points were filled in. After measurements, the maximum ratio of non-measured points was 15%. The spikes were removed by surface truncation to material ratios between 0.13 and 99.87%. Digital filtration was not used.

The application of a tip radius of 2 µm does not cause the distortion of the results of surface topography measurements [1,2]. In workshops, the use of stylus tip radii of 5 µm and 10 µm is common. The distortions surface texture using these radii can be high but have not been examined comprehensively yet. The radius of the stylus tip cannot be smaller than the sampling interval used (3.29 µm). The application of a 5× objective lens allows the measurement of a comparatively large surface area. Therefore, mechanical filtrations by the stylus tip of radii 5 and 10 µm were simulated using morphological dilation operations. During a dilation, the structuring element, being a circular disc, is in contact with and above the surface. This operation is equivalent to an envelope. The stylus tip material was assumed to be rigid. The method of mechanical filtration simulation using the dilation procedure was validated using surfaces with modelled triangular grooves [38].

The areal (3D) surface texture parameters were calculated using the TalyMap 6 software program according to the ISO 25178:2 standard. The following height parameters were used: arithmetical mean of absolute surface heights (Sa), rms. height (Sq), skewness (Ssk), and kurtosis (Sku). Skewness and kurtosis were described in [47]. The following were also analysed: spatial parameters (correlation length (Sal) and texture aspect ratio (Str)), hybrid parameters (rms. slope (Sdq) and the developed interfacial areal ratio (Sdr)). Among the features, peak density (Spd) and the mean peak curvature (Spc) were studied. Definitions and interpretations of the parameters analysed are presented in References [48,49]. The amplitude parameters often decide on tribological properties of machine parts; they are also used in the quality assessment of machine elements. Skewness (Ssk) is also important in tribological problems; its negative values are recommended because they lead to the enhanced contact of surfaces and frequently to small wear and friction. The Sdr parameter is important in the creation of an adhesive joint, coating adhesion, and corrosion protection [1,2,48]. The surface slope affects the tribological properties; it is also related to light reflection. Spatial parameters are substantial in lubrication. Peak density and peak curvature decide on the contact of rough surfaces [48].

In addition, three parameters related to furrows were analysed: maximum depth of furrows, mean depth of furrows, and mean density of furrows. These parameters are important in dermatology. The density of the furrows decreased with age [50], while the depth of furrows increased with age [51,52]. The photoexposed sites showed more linear, distant, and thin furrows than the photoprotected sites [53].

The number of parameters analysed was 13. Relative average and maximum parameter changes calculated in relation to the results of surface topography measurement using a white light interferometer after form removal, filling in non-measured points, and spike removal will be presented.

A total of 15 surfaces were anisotropic (the texture aspect ratios (Str) were smaller than 0.15); 15 surfaces had a mixed character (the Str values were between 0.15 and 0.84); and other surfaces were isotropic (the Str values were more than 0.84).

## 3. Results and Discussion

Anisotropic surfaces are characterised by very low Str parameter values. Two-directional surfaces after grinding and milling and cross-hatched surfaces after one-process and two-process honing (plateau honing) were analysed (15 surfaces). Table 2 presents the average and relative changes in the absolute values of the areal texture parameters of the anisotropic surfaces.

The height parameters decreased due to mechanical filtration by a stylus tip, and the changes in Sq were a little higher than those of Sa. Among parameters characterising the shape of the ordinate distribution, the skewness (Ssk) typically increased, especially for the surfaces of symmetric ordinate distributions, but no direct tendency of the Sku parameter was found. The high relative changes in the Ssk parameter resulted from its value near zero. The spatial parameters increased mainly as a result of mechanical filtration. Sometimes, high changes in the Str parameter were related to very small values of this parameter of anisotropic surfaces. Hybrid parameters decreased as a result of mechanical filtration by the radius of the tip of the stylus. The decrease in the Sdr parameter caused by the increase in the radius of the tip of the stylus was greater than that of the rms. slope (Sdq). High relative changes in hybrid parameters were caused by the height decrease and increase in the main wavelength, characterised by the correlation length (Sal). The peak density (Spd) decreased, which is connected with the growth in the Sal parameter. The average peak curvature (Spc) decreased, and its changes were comparatively high. The density and depth of the furrows mainly decreased as the radius of the tip of the stylus increased. The changes in the density of the furrows were the smallest.

The increase in the tip of the radius of the stylus caused higher errors in determining surface texture parameters. Among the fundamental parameters (amplitude and spatial), the mechanical filtration of the stylus tip typically led to a decrease in surface amplitude and an increase in the spatial parameters Sal and Str. The growth in the Sal caused a decrease in the peak density (Spd). These changes are typical for low-pass digital filtration. They caused very high changes in the hybrid parameters Sdq and Sdr and the mean peak curvature (Spc). Changes in parameters caused by the impact of the stylus tip radius were smaller for lower amplitudes and higher main wavelengths. For the analysed anisotropic textures, parameter changes caused by digital filtration were the smallest for surfaces after fine milling. Mechanical filtration by the tip of the stylus caused small changes in maximum amplitude. Scanning orientation does not play a role in the distortion of anisotropic surfaces.

**Figure 2:**
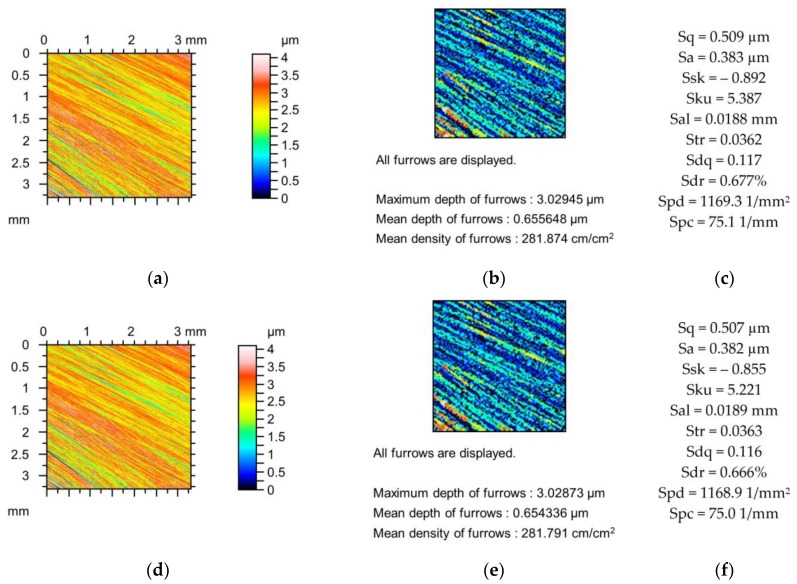

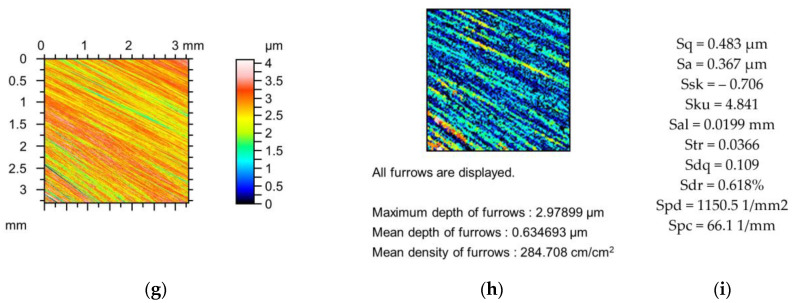
Contour plots (**a**,**d**,**g**), view of furrows (**b**,**e**,**h**) and texture parameters (**c**,**f**,**i**) of ground surface after measurement (**a**–**c**), and simulated mechanical filtration by the tip of the stylus (by dilation procedure) of radius 5 µm (**d**,**e**,**g**) and 10 µm (**g**–**i**).

**Figure 3:**
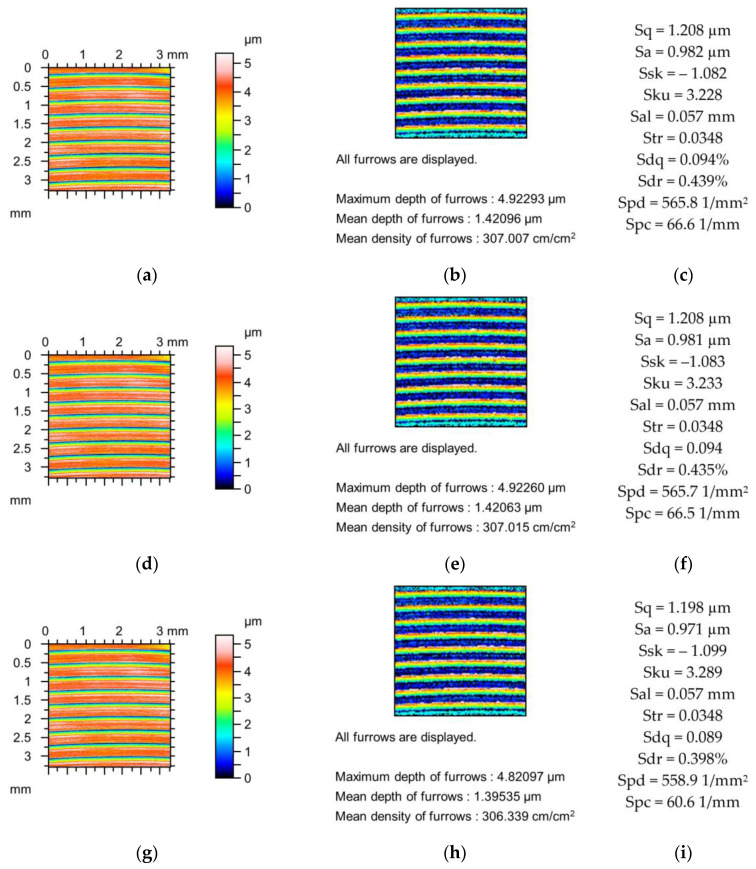
Contour plots (**a**,**d**,**g**), view of furrows (**b**,**e**,**h**) and texture parameters (**c**,**f**,**i**) of the milled surface after measurement (**a**–**c**), and simulated mechanical filtration by the tip of the stylus (by dilation procedure) of radius 5 µm (**d**,**e**,**g**) and 10 µm (**g**–**i**).

**Figure 4:**
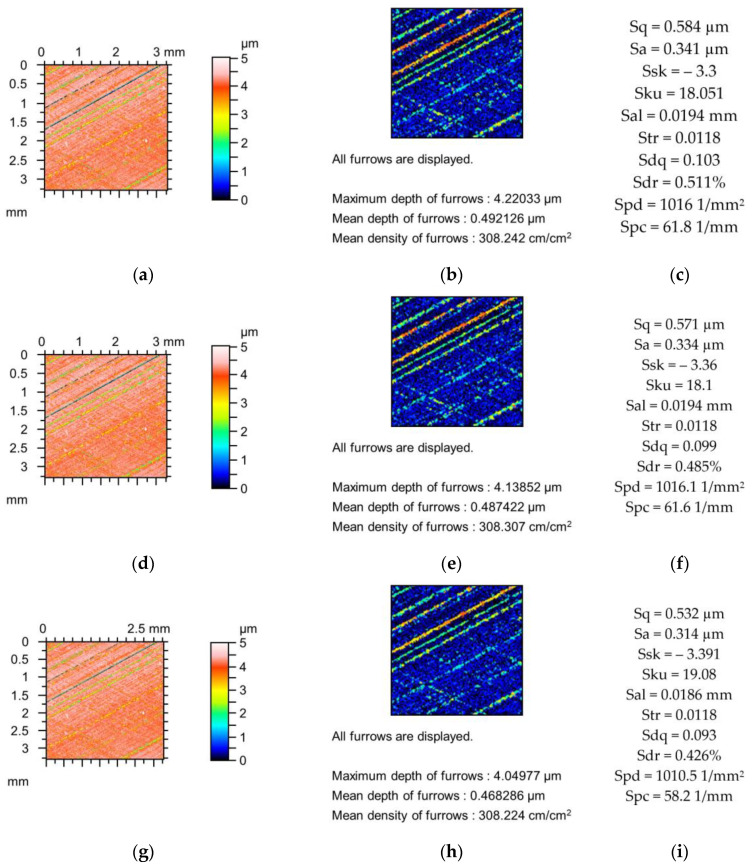
Contour plots (**a**,**d**,**g**), view of furrows (**b**,**e**,**h**) and texture parameters (**c**,**f**,**i**) of plateau honed surface after measurement (**a**–**c**), and simulated mechanical filtration by the stylus tip (by dilation procedure) of radius 5 µm (**d**,**e**,**g**) and 10 µm (**g**–**i**).

Figure 2, Figure 3 and Figure 4 present changes in the contour plots, furrows, and parameters for surfaces after grinding, milling, and plateau honing, respectively.

The decreases in the amplitude parameters of the ground surface shown in Figure 2 were up to 5%. Skewness (Ssk) increased and kurtosis (Sku) decreased to 10%. Spatial parameters increased (Sal to 6% and Str to 1%), and the peak density (Spd) decreased to 2%. The reductions in the hybrid parameters Sdq and Sdr and the peak density (Spc) were up to 6, 9, and 12%, respectively. The decreases in the depths of the furrows caused by changes in the tip radii were smaller than 3%, and the changes in the furrow density were not higher than 5% due to the simulated mechanical filtration by the stylus tip.

The changes in the parameters of the milled surface shown in Figure 3 due to the simulated mechanical filtration by the stylus tip were smaller compared to those of the previously analysed ground surface (Figure 2) of similar maximum height. The maximum reduction in the amplitude parameters was 1%; Sku increased to 2%; Sal increased to 5%; Str was constant; the peak density decreased to 1%; and parameters Sdq, Sdr, and Spc decreased to 5, 9, and 9%, respectively. The reductions in the depths of the furrows were less than 2%, and the density of furrows decreased to 1%. These comparatively low changes were probably caused by the higher correlation length (Sal) of the milled texture compared to the ground surface, as shown in Figure 2.

Changes in the parameters of the plateau-honed cylinder surface due to simulated mechanical filtration with a stylus tip are shown in Figure 4. The amplitude parameters decreased to 9%, the skewness decreased, and the kurtosis increased to 6%. Sal and Str increased to 4 and 1%, respectively, and the peak density decreased to 0.5%. The maximum decreases in parameters Sdq, Sdr, and Spc were 5, 16, and 7%, respectively. The depths of the furrows decreased to 4%, and changes in the densities of the furrows were negligible.

Surfaces after polishing and lapping, as well as textured surfaces with isolated oil pockets, belong to mixed textures. Dimples were created by burnishing or laser treatment, and surface details between oil pockets were created by grinding or lapping. Table 3 presents the average and relative changes in the absolute values of texture parameters of mixed 15 surfaces. The decreases in the amplitude parameters due to mechanical filtration by a stylus tip were similar to anisotropic surfaces (Table 2). The changes in other parameters of mixed surfaces were smaller than those of anisotropic textures. The small average changes were due to the fact that impacts from stylus tips did not affect surfaces after polishing, for which the maximum surface height was less than 1 µm. The smaller relative changes in the Str and Ssk parameters were caused by the higher values of these parameters of textured surfaces compared to those of anisotropic surfaces. Similarly to the anisotropic surfaces, the highest changes in the Spc and Sdr parameters occurred among the parameters analysed. These parameters decreased as a result of mechanical filtration. The peak density (Spd) also decreased. The changes in the spatial parameters were smaller than those in the amplitude parameters. In most cases, the skewness (Ssk) and the kurtosis (Sku) increased. No clear tendencies of the changes in the Sal and Str parameters were found. The mean depth of the furrows decreased owing to mechanical filtration; tendencies of changes in other parameters characterising the furrows were not found. The increase in the simulated tip radius caused greater changes in the surface texture parameters. Generally, mixed textures showed a low sensitivity to tip radius despite their geometrical complexity due to their comparatively small roughness height.

**Figure 5:**
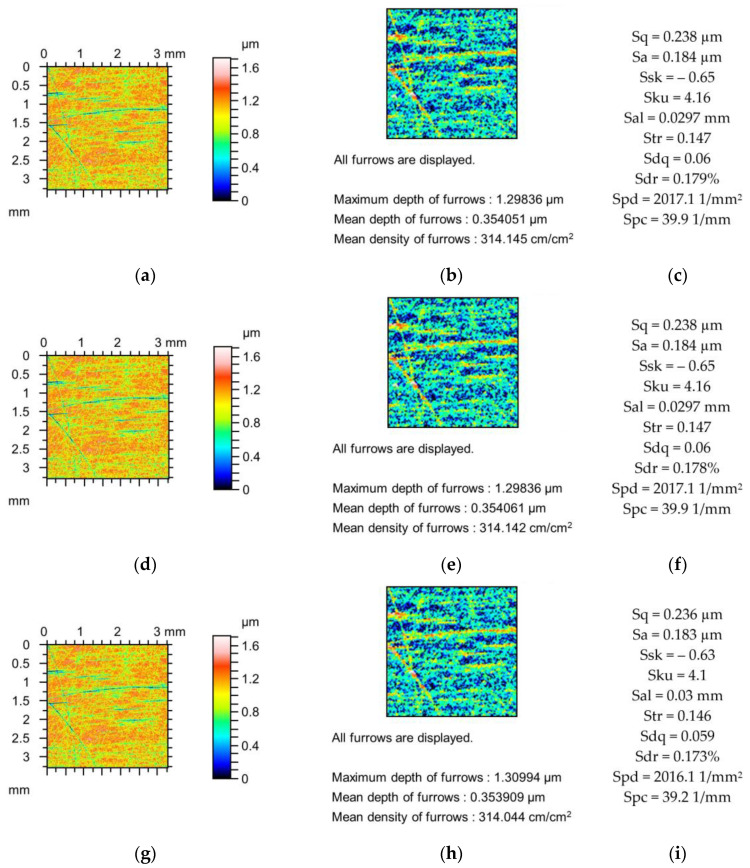
Contour plots (**a**,**d**,**g**), view of furrows (**b**,**e**,**h**) and texture parameters (**c**,**f**,**i**) of the lapped surface after measurement (**a**–**c**), and simulated mechanical filtration by the tip of the stylus (by dilation procedure) of radius 5 µm (**d**,**e**,**g**) and 10 µm (**g**–**i**).

**Figure 6:**
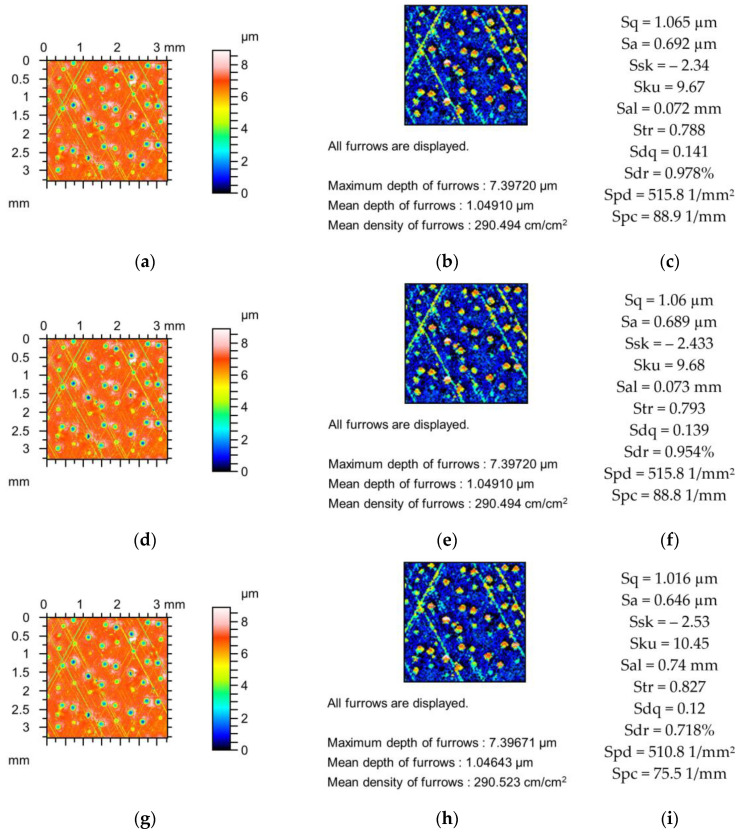
Contour plots (**a**,**d**,**g**), view of furrows (**b**,**e**,**h**) and texture parameters (**c**,**f**,**i**) of the textured surface using burnishing after measurement (**a**–**c**), and simulated mechanical filtration by the tip of the stylus (by dilation procedure) of radius 5 µm (**d**,**e**,**g**) and 10 µm (**g**–**i**).

**Figure 7:**
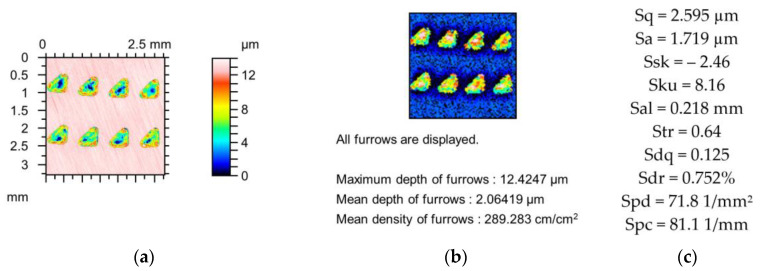

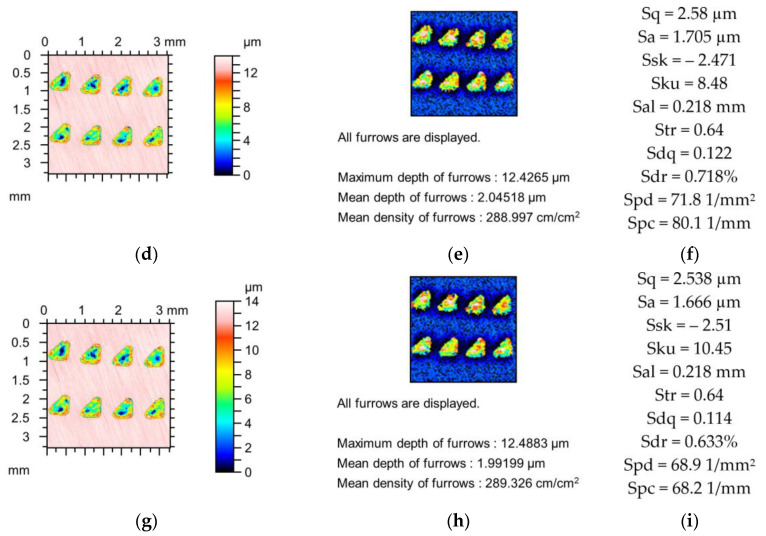
Contour plots (**a**,**d**,**g**), view of the furrows (**b**,**e**,**h**) and texture parameters (**c**,**f**,**i**) of the laser textured surface after measurement (**a**–**c**), and simulated mechanical filtration by the tip of the stylus (by dilation procedure) of radius 5 µm (**d**,**e**,**g**) and 10 µm (**g**–**i**).

Figure 5, Figure 6 and Figure 7 present changes in the contour plots, furrows, and parameters for surfaces after lapping, texturing using burnishing, and laser texturing, respectively.

The effect of a stylus tip radius of 5 µm caused a marginal decrease in the only Sdr parameter of surface after lapping (Figure 5). The higher increase in the tip of the radius of the stylus caused the highest decrease in the Sdr parameter (3.3%) followed by the Spc parameter (2.5%). The relative changes in the parameters Ssk, Sku, and Sdq were between 2 and 3%. The variations in other parameters were smaller. The low number of changes in the parameters caused by mechanical filtration were caused by the low surface amplitude.

The relative changes in the parameters of the cylinder surface with oil pockets created by the burnishing technique (Figure 6) were much higher than those of the lapped surface, as shown in Figure 5. The largest change in the Sdr parameter of approximately 3% was found for the application of a stylus tip radius of 5 µm. Variations in other parameters were less than 1%. Higher changes were obtained for a stylus tip radius of 10 µm. The highest decrease was found in the Sdr parameter (27%) followed by Spc and Sdq (15%). The surface amplitude was reduced to 7%, while Ssk decreased and Sku increased to 4 and 8%, respectively. The spatial parameters Str and Sal increased to 3 and 5%, respectively. A low reduction in the peak density (Spd) of approximately 1% was found. The maximum and mean depth of the furrows decreased by 3 and 5%, respectively. A negligible change in the density of the furrows was found.

Similar changes occurred for other textured surfaces shown in Figure 7. The texturing of the steel surface was performed using laser treatment. The tip radius of 5 µm caused the highest reduction in the Sdr parameter (4%) followed by Sdq (2%). When the radius of the stylus tip increased to 10 µm, the greatest reductions were found for parameters Sdr and Spc (16) followed by Sdq (9%). The surface amplitude decreased to 3.5%, while the skewness (Ssk) decreased and the kurtosis (Sku) increased to 2 and 4%, respectively. The peak density (Spd) decreased to 4%, but the spatial parameters Str and Sal did not change. The mean depth of furrows decreased to 3.5%, but the changes in other parameters characterising furrows were negligible.

Table 4 presents the average and relative changes in the absolute values of the parameters of isotropic surfaces. One-process surfaces (with traces of one machining process, after vapour blasting) and two-process surfaces (after vapour blasting followed by lapping) were analysed. Two-process surfaces were characterised by smaller amplitudes and asymmetric ordinate distributions, described by comparatively high negative values of skewness. Similarly to the surfaces analysed above, the amplitudes of surface textures decreased due to the mechanical filtration by the stylus tip. The changes in the mean amplitude parameters Sa and Sq were similar. In most cases, the skewness (Ssk) decreased for two-process surfaces and increased for one-process textures. The kurtosis (Sku) usually increases. The relative changes in kurtosis were smaller than those in skewness. Mechanical filtration by the tip of the stylus caused an increase in the correlation length Sal for all surfaces analysed. Growth in the correlation length is related to reduction in the peak density (Spd). The changes in amplitude and autocorrelation length were the highest for all surface types analysed. The tendency of changes in the Str parameter was not clear. Comparatively small relative deviations resulted from high values of this parameter for isotropic surfaces. The reduction in amplitude and growth in the correlation length caused high decreases in the values of hybrid parameters and the peak density (Spd). Similarly to other types of textures, the changes in Sdr and Spc were greater than those in Sdq. The densities of the furrows and mean depths of the furrows decreased, and relative changes in the average depths were higher than those of the densities. No clear trends of changes in maximum depth of the furrows were found.

**Figure 8:**
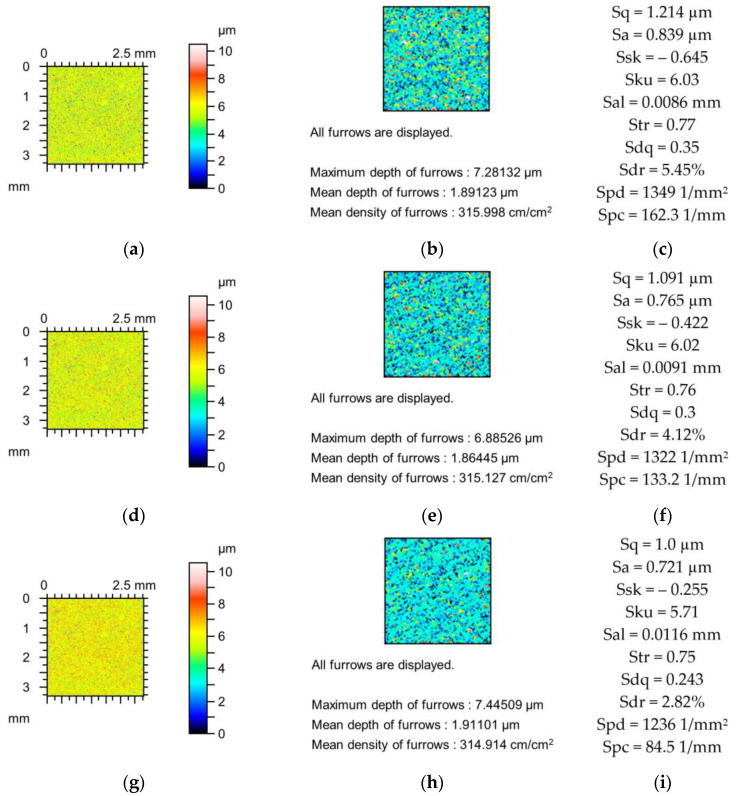
Contour plots (**a**,**d**,**g**), view of the furrows (**b**,**e**,**h**) and texture parameters (**c**,**f**,**i**) of the vapour-blasted surface after measurement (**a**–**c**), and simulated mechanical filtration by the tip of the stylus (by dilation procedure) of radius 5 µm (**d**,**e**,**g**) and 10 µm (**g**–**i**).

Figure 8 presents changes in the contour plot, furrow, and parameters of surfaces after the vapour blasting caused by the action of the stylus tip. Changes in parameters occurred for both radii of the stylus tip. The decreases in the amplitude parameters were not greater than 18%. Skewness (Ssk) increased and kurtosis decreased marginally. The correlation length (Sal) increased to 35%, but the decrease in the (Str) parameter was much smaller. The peak density (Spd) decreased to 8%. A high decrease in the hybrid parameters Sdq and Sdr and the mean peak curvature (Spc) occurred, and the maximum changes were 30.5, 48, and 48%, respectively.

**Figure 9:**
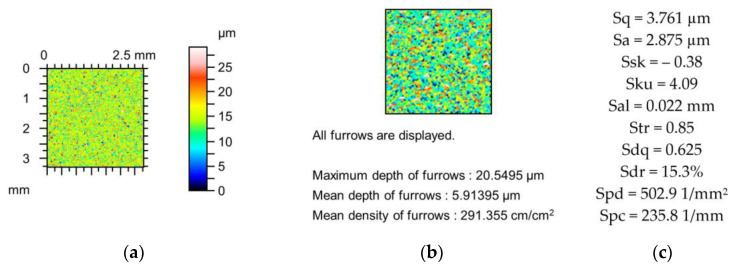

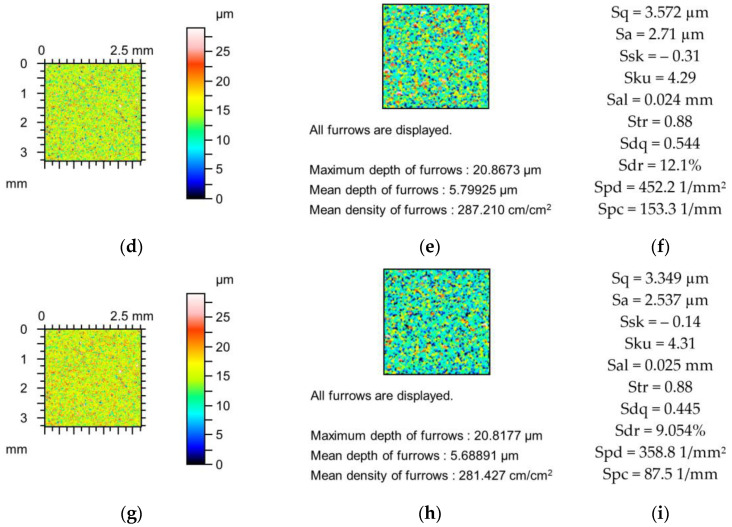
Contour plots (**a**,**d**,**g**), view of the furrows (**b**,**e**,**h**) and texture parameters (**c**,**f**,**i**) of vapour blasted surface after measurement (**a**–**c**), and simulated mechanical filtration by the tip of the stylus (by dilation procedure) of radius 5 µm (**d**,**e**,**g**) and 10 µm (**g**–**i**).

Changes in other surface texture after vapour blasting caused by the radii of the stylus tip are presented in Figure 9. This surface is characterised by a higher amplitude and greater correlation length than the surface shown in Figure 8. The changes in the parameters of these two surfaces are similar. The surface amplitude shown in Figure 9 decreased by not more than 11%. Skewness and kurtosis increased. Changes in kurtosis (Sku) were approximately 5%. The correlation length (Sal) increased to 12%, and the Str parameter increased marginally. The peak density (Spd) decreased to 28%. Parameters Sdq, Sdr, and Spc were reduced, and their maximum changes were 29, 41, and 63%, respectively.

**Figure 10:**
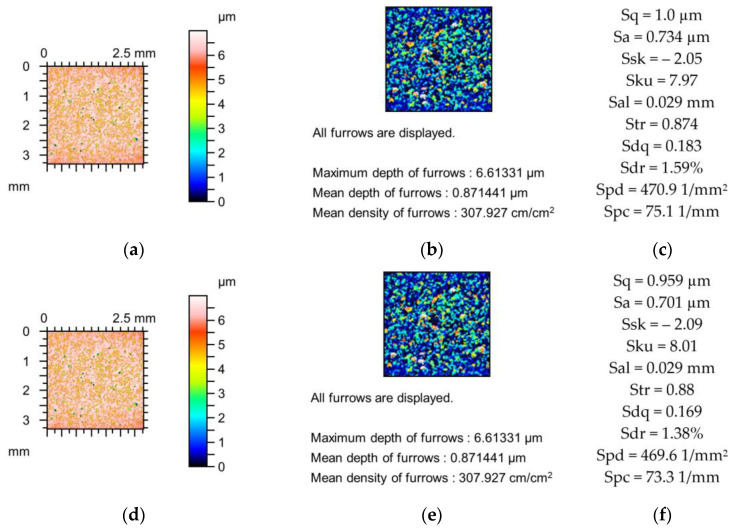

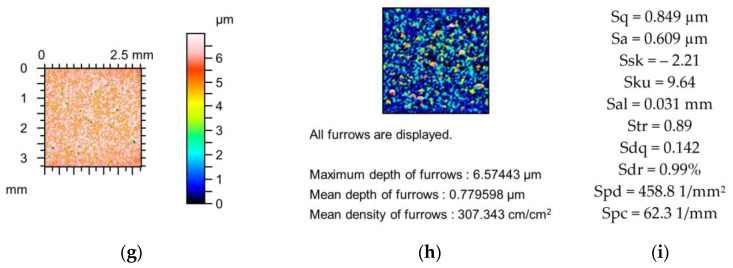
Contour plots (**a**,**d**,**g**), view of furrows (**b**,**e**,**h**) and texture parameters (**c**,**f**,**i**) of vapour blasted and lapped surface after measurement (**a**–**c**), and simulated mechanical filtration by the tip of the stylus (by dilation procedure) of radius 5 µm (**d**,**e**,**g**) and 10 µm (**g**–**i**).

Figure 10 presents changes in the contour plot, the furrow, and the parameters of two-process surfaces after a vapour blasting followed by lapping caused by simulated application of the stylus tip. This type of surface is characterised by a negative skewness value (smaller than −1). The amplitude parameters decreased to 17%. Skewness (Ssk) decreased, which is a characteristic feature of the two-process surfaces. Kurtosis (Sku) increased to 21%. Peak density (Spd) decreased to 2.5%. The hybrid parameters Sdq and Sdr decreased to 22 and 38%, respectively. The decrease in the mean peak curvature (Spc) was comparatively small and amounted to 17%.

The analysis of the stylus tip size impact was performed using simulation. The dilation procedure was used. Simulation facilitates a decrease in the time and cost of experimental investigations. It also eliminates the effects of other factors influencing measurement errors, such as measurement speed, thermal disturbances, surface deformation, and high-frequency noise [27,28,29,30,31,32,33]. The effect of mechanical filtration with the stylus tip on variations in areal surface texture parameters was analysed for various types of surfaces (anisotropic, mixed, or isotropic) to obtain the tendencies of surface texture parameter changes. This study was conducted to eliminate other possible sources of deviations in the results, for example, due to the sampling interval, measurement area, and reference element. Typically, the effect of the size of the stylus tip on the changes in amplitude parameters was studied [1,2,36,37,38]. However, not only the amplitude parameters can be changed due to mechanical filtration. In this work, the effects of 5 and 10 µm radii were analysed. In laboratory works, a tip radius of 2 µm is commonly used. However, radii of 5 and 10 µm are also admissible. Substantial distortions of smooth profiles can be high [36,37], but were not examined comprehensively in three dimensions. In addition, the radius of the stylus tip due to wear can be increased [1,2].

In this work, the relative changes in the selected surface texture parameters due to mechanical filtration by the stylus tip were studied. These deviations depend on the values of the parameters and can be high when these values are close to 0. This is particularly important for Str and Ssk. The error in determining the Str parameter can be high for anisotropic surfaces for which the Str parameter is close to 0. Similarly, the relative deviation of the skewness (Ssk) can be great for a one-process surface of approximately symmetric ordinate distribution, for which this parameter is close to 0.

Regardless of surface type, an increase in the radius of the stylus tip caused larger changes in the areal surface texture. For all types of surfaces, mechanical filtration caused reductions in the parameters Sa and Sq. A reduction in roughness height was also found in References [36,37,38,39,40,41]. The changes in the parameters that describe the shape of the height distribution, Ssk and Sku, depended on the type of surface. For a one-process surface of an approximately symmetric shape to that of the ordinate distribution, the skewness (Ssk) increased, but for two-process surfaces with initially negatively skewness, it typically decreased as a result of mechanical filtration (Figure 5, Figure 6, Figure 7 and Figure 10). For one-process textures, perhaps the stylus tip did not touch the bottom of narrow valleys, so the skewness increased as a result of mechanical filtration. However, deep valleys were wide, so their changes were marginal due to mechanical filtration by the stylus tip, and therefore, only changes in small (non-deep) valleys occurred and skewness decreased. The situation can be changed for narrow deep valleys. For two-process surfaces, the kurtosis increased as a result of mechanical filtration by a stylus tip. Typically, smaller values of negative skewness correspond to higher values of kurtosis [47,48] (Figure 5, Figure 6, Figure 7 and Figure 10). For one-process surfaces, a non-clear tendency of kurtosis changes was found.

For anisotropic and isotropic surfaces, mechanical filtration by the stylus tip caused an increase in the correlation length (Sal). Similar changes were also found in Reference [38]. When a stylus tip device is used, high-frequency components are filtered out [40,41]. No clear changes in the correlation length were found for mixed surfaces with generated oil pockets. However, for this type of surface, the oil pockets decided on the autocorrelation function and consequently on the Sal parameter (these changes were very small). The increase in the correlation length (Sal) resulted in a reduction in the peak density Spd. A reduction in the peak density was also found for mixed surfaces with isolated oil pockets; this observation suggests that the mean wavelength for the peak part also increased as a result of mechanical filtration. The Str parameter of the anisotropic surfaces increased as a result of mechanical filtration by the stylus tip. This behaviour resulted from the definition of the Str parameter, which is the ratio of the smallest and highest correlation lengths. Due to the impact of the radius of the stylus tip, the smallest correlation length (Sal) increased, but the highest correlation length was constant; therefore, the Str parameter increased. For other types of surfaces, no clear tendency of changes in the Str parameter due to mechanical filtration was found.

In all cases analysed, the hybrid parameters Sdq and Sdr and the Spc feature parameter were reduced because of mechanical filtration. These reductions were the highest for anisotropic and isotropic textures and resulted from the decrease in the roughness amplitude and the growth in the correlation length (Sal). The changes in Sdr and Spc were higher than those in Sdq. Decreases in hybrid parameters due to mechanical filtration were also found in References [38,39]. The Sdr parameter is characterised by a high sensitivity to measurement errors [6], so the Sdq parameter should be preferred over hybrid parameters.

Changes in parameters that characterise furrows depend on the surface type. For anisotropic surfaces, the density and depth of furrows typically decreased. The average depth of the furrows was reduced when the mixed surfaces were mechanically filtered, and changes in other parameters characterising the furrows were not clear. For isotropic surfaces, densities of furrows and mean depths of furrows decreased, whereas non-clear tendency of changes in maximum depth of furrows was obtained. For all types of surfaces, the highest changes (reductions) occurred for the mean depth of the furrows. Generally, the depth and density of the furrows had a tendency to decrease as a result of mechanical filtration by the tip of the stylus.

The results of surface texture measurements were more distorted by mechanical filtration of the stylus tip for higher roughness amplitudes and shorter wavelengths. Typically, smoother surfaces were characterised by smaller spatial parameters such as the correlation length. However, in this research, no changes in parameters were found for smooth polished and lapped surfaces. The ratio of the highest to smallest roughness amplitudes determined by the Sq parameter was higher than the ratio of the largest and smallest correlation lengths. Therefore, the impact of roughness height seemed to be greater than that of the main wavelength. However, due to the sampling interval used and the optical method of measurement, the minimum wavelengths of the smooth surfaces could be filtered out.

**Figure 11:**
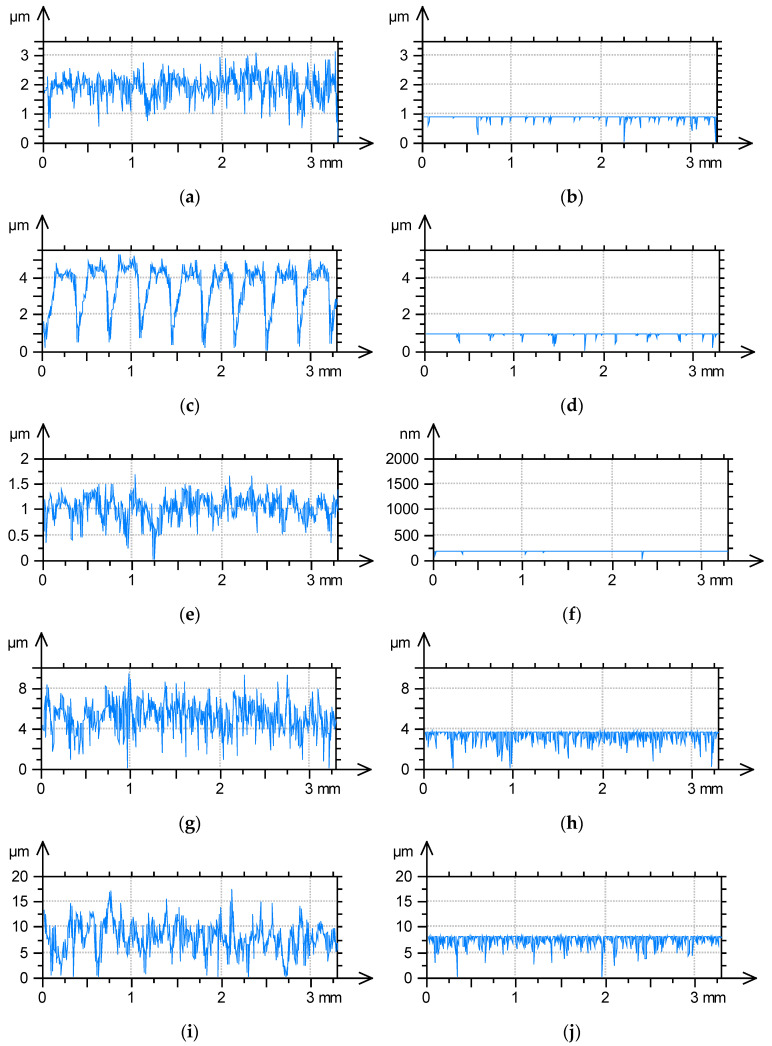
Extracted profiles (**a**,**c**,**e**,**g**,**i**) and removed profiles as the result of simulated mechanical filtration with a stylus tip of a radius of 10 µm radius (**b**,**d**,**f**,**h**,**j**) of surfaces shown in Figure 2a, Figure 3a, Figure 5a, Figure 8a and Figure 9a.

Figure 11 presents the extracted profiles and the removed profiles as the result of mechanical filtration with a stylus tip of 10 µm radius for the selected surface textures. The change in surface texture after milling (Figure 3) is greater than that of the surface after grinding (Figure 2) of similar maximum roughness height. This difference was caused by the longer correlation length of the milled surface. This observation was confirmed by the analysis of the profile changes shown in Figure 11a–d. The removed profile of the ground surface (Figure 11b) contains more scratches than that of the milled surface (Figure 11d). An analysis of Figure 11e,f confirmed small changes in surface texture after lapping, as shown in Figure 5. The removed profile in Figure 11f contains only five valleys. Figure 11g–j correspond to the surfaces after vapour blasting shown in Figure 8 and Figure 9, respectively. The surface texture in Figure 8 had a lower roughness height and a shorter correlation length than the surface in Figure 9. Therefore, the changes in these surface textures due to mechanical filtration by stylus tip were rather similar. This observation was confirmed by the analysis of the profiles in Figure 11g–j. The removed profile shown in Figure 11h is characterised by a smaller maximum amplitude but more dense valleys (smaller main wavelength) than the profile in Figure 11j.

The results of simulated mechanical filtration by the stylus tip were dependent on the method of spike removal. When it was not used, the relative changes in the amplitude parameters, including the maximum roughness height, were higher compared to those obtained after the elimination of the spikes.

For precise surface texture measurement in scientific applications, the radius of the stylus tip should be smaller than 5 µm. Due to possible wear, stylus tip radius should be often checked. For comparative roughness measurement in industry applications, a 5 µm tip radius can be admitted.

This work focusses on the effect of mechanical filtration of the stylus tip on changes in the areal parameter of surface texture measurement. However, other factors also caused errors in texture measurement, such as thermal disturbances, high-frequency noise, scanning speed, stylus tip wear, and the plastic deformation of the measured surface.

## 4. Conclusions

1. The size of the stylus tip affects the results of areal surface texture measurement. This influence is similar to low-pass filtering and is therefore called mechanical filtration. The increase in the radius of the stylus tip caused greater changes in surface texture.
2. Regardless of the type of surface, mechanical filtration with a stylus tip caused a reduction in the mean amplitude parameters Sa and Sq. The changes in these two parameters were similar.
3. The changes in skewness (Ssk) and kurtosis (Sku) due to mechanical filtration by the stylus tip depended on the type of surface. For one-process surfaces, the skewness (Ssk) increased, but for two-process textures, it typically decreased as a result of mechanical filtration. For two-process surfaces, the kurtosis (Sku) increased due to mechanical filtration by a stylus tip. For one-process textures, no tendency of kurtosis changes was found.
4. During mechanical filtration by the stylus tip, low wavelengths were filtered out. In most cases, the correlation length increased. The increase in the correlation length (Sal) resulted in a decrease in the peak density (Spd).
5. The Str values of anisotropic surfaces increased as a result of mechanical filtration, which is related to the increase in the Sal parameter. For other types of surfaces, the tendency of changes in the Str parameter was not found.
6. The hybrid parameters Sdq and Sdr decreased as a result of mechanical filtration by a stylus tip. Changes in Sdr were higher than those of Sdq, and therefore, the Sdq parameter is preferred. A comparative high decrease in the Spc parameter was also found. Changes in the Sdq, Sdr, and Spc parameters were in most cases caused by decreasing the amplitude and increasing the correlation length.
7. The depth and density of the furrows had a tendency to decrease as a result of mechanical filtration by the stylus tip. The highest changes occurred for the mean depth of furrows.
8. The results of surface texture measurement are more distorted during measurement with a stylus tip device for higher roughness amplitude and a smaller main wavelength. The impact of the roughness height was higher than that of the wavelength.
9. The proposed model of mechanical filtration by the stylus tip does not consider other factors affecting the results of surface topography measurement such as scanning speed, tip wear, surface deformation, thermal disturbances, and high-frequency noise.

## Figures and Tables

**Table 1:** Specifications of measured samples.

Machining Process	Material	Number of Samples
grinding	steel	4
milling	steel	4
one-process honing	grey cast iron	3
plateau honing	grey cast iron	4
polishing	steel	4
lapping	steel	4
texturing using burnishing	grey cast iron	3
laser texturing	steel	4
vapour blasting	steel	8
vapour blasting and lapping	steel	7

**Table 2:** Average and relative changes in absolute values of texture parameters of anisotropic surfaces caused by stylus tip radii of 5 and 10 µm, mechanical filtration was simulated by dilation.

Parameter	Stylus Tip Radius 5 µm		Stylus Tip Radius 10 µm	
	Average Changes, %	Maximum Changes, %	Average Changes, %	Maximum Changes, %
Sq	0.9	2.4	2.7	8.7
Sa	0.8	2.4	2.6	6.7
Ssk	1.9	13.1	4.6	12.1
Sku	0.7	3.1	1.3	9.5
Str	2.2	18.1	3.3	19.1
Sal	0.4	1.5	1.6	5.8
Sdq	2.9	8.5	9.1	17.9
Sdr	5.8	17.1	15.3	33.6
Spd	0.3	1.3	4.8	14.3
Spc	6.6	39.1	18.2	63.3
Density of furrows	0.05	0.5	0.43	1.3
Maximum depth of furrows	0.54	2.1	2.1	6.8
Mean depth of furrows	0.9	3.5	2.7	13.1

**Table 3:** Average and relative changes in absolute values of texture parameters of mixed surfaces caused by stylus tip radii of 5 and 10 µm; mechanical filtration was simulated by dilation.

Parameter	Stylus Tip Radius 5 µm		Stylus Tip Radius 10 µm	
	Average Changes, %	Maximum Changes, %	Average Changes, %	Maximum Changes, %
Sq	0.9	4.4	2.7	10.7
Sa	0.7	2.7	2.6	7.4
Ssk	0.5	2.1	1.4	5.1
Sku	0.9	4.5	2.6	10.5
Str	0.02	0.1	0.6	5.1
Sal	0.2	1.1	0.9	2.8
Sdq	0.7	2.4	4.9	14.8
Sdr	2.3	6.6	8.4	27.5
Spd	0.3	2.9	1.2	5.7
Spc	2.1	17.2	8.9	41.1
Density of furrows	0.07	0.4	0.16	0.7
Maximum depth of furrows	0.05	0.4	0.7	2.7
Mean depth of furrows	0.4	1.5	2.6	6.4

**Table 4:** Average and relative changes in absolute values of texture parameters of isotropic surfaces caused by stylus tip radii of 5 and 10 µm; mechanical filtration was simulated by dilation.

Parameter	Stylus Tip Radius 5 µm		Stylus Tip Radius 10 µm	
	Average Changes, %	Maximum Changes, %	Average Changes, %	Maximum Changes, %
Sq	5.9	9.6	13.7	17.5
Sa	5.7	8.9	13.4	18.3
Ssk	12.9	37.1	28.8	65.1
Sku	2.65	7.9	8.6	22.1
Str	1.15	2.8	1.31	4.3
Sal	4.38	13.7	12.2	39.7
Sdq	13.1	18.2	26.7	34.5
Sdr	21.7	36.1	42.3	53.1
Spd	2.53	3.9	17.1	30.3
Spc	21.5	43.1	43.2	63.1
Density of furrows	0.78	2.4	1.48	2.4
Maximum depth of furrows	1.44	4.9	2.12	5.4
Mean depth of furrows	2.6	6.4	7.3	17.8

## Data Availability

The original contributions presented in the study are included in the paper, and further inquiries can be directed to the corresponding author.

## References

[1] Whitehouse D.J. (1994). Handbook of Surface Metrology.

[2] Thomas T.R. (1999). Rough Surfaces.

[3] Korn V., Surber C., Imanidis G. (2016). Skin Surface Topography and Texture Analysis of Sun-Exposed Body Sites in View of Sunscreen Application. Skin Pharmacol. Physiol..

[4] Pang H., Chen T., Wang X., Chang Z., Shao S., Zhao J. (2017). Quantitative Evaluation Methods of Skin Condition Based on Texture Feature Parameters. Saudi J. Biol. Sci..

[5] Moon C.I., Lee O. (2018). Age-Dependent Skin Texture Analysis and Evaluation Using Mobile Camera Image. Skin Res. Technol..

[6] Pawlus P., Reizer R., Wieczorowski M., Krolczyk G. (2024). Sensitivities of Surface Texture Parameters to Measurement Errors—A Review. Measurement.

[7] (2010). Geometrical Product Specifications (GPS)—Surface Texture: Areal—Part 6: Classification of Methods for Measuring Surface Texture.

[8] (2010). Geometrical Product Specifications (GPS)—Surface Texture: Areal—Part 601: Nominal Characteristics of Contact (Stylus) Instruments.

[9] Nayak P.R. (1971). Random Process Model of Rough Surfaces. J. Lubr. Technol..

[10] Jiang X., Blunt L. (2003). Advanced Techniques for Assessment Surface Topography.

[11] Stout K.J., Blunt L. (2000). Three-Dimensional Surface Topography.

[12] de Groot P., de Lega X.C., Su R., Leach R. (2019). Does Interferometry Work? A Critical Look at the Foundations of Interferometric Surface Topography Measurement. Appl. Opt. Metrol. III.

[13] Leach R.K. (2011). Optical Measurement of Surface Topography.

[14] Whitehouse D.J. Surface metrology today: Complicated, confusing effective?. Proceedings of the 13th International Conference on Metrology and properties of Engineering Surfaces.

[15] Li Z., Gröger S. (2022). Experimental Study of Non-Measured Points on Surface Measurement Using Structured Illumination Microscopy. Metrol. Meas. Syst..

[16] Pawlus P., Reizer R., Wieczorowski M. (2017). Problem of Non-Measured Points in Surface Texture Measurements. Metrol. Meas. Syst..

[17] Podulka P., Pawlus P., Dobrzański P., Lenart A. (2014). Spikes Removal in Surface Measurement. J. Phys. Conf. Ser..

[18] Li Z., Gröger S. (2021). Investigation of Noise in Surface Topography Measurement Using Structured Illumination Microscopy. Metrol. Meas. Syst..

[19] Gomez C., Su R., de Groot P., Leach R. (2020). Noise Reduction in Coherence Scanning Interferometry for Surface Topography Measurement. Nanomanufacturing Metrol..

[20] Lei Z., Liu X., Zhao L., Chen L., Li Q., Yuan T., Lu W. (2016). A Novel 3D Stitching Method for WLI-Based Large Range Surface Topography Measurement. Opt. Commun..

[21] Makki S., Barbenel J.C., Agache P. (1979). A Quantitative Method for the Assessment of the Microtopography of Human Skin. Acta Derm. Venereol..

[22] Kautzky F., Dahm M.W., Drosner M., Köhler L.D., Vogt H.J., Borelli S. (1995). Direct Profilometry of the Skin: Its Reproducibility and Variability. J. Eur. Acad. Dermatol. Venereol..

[23] Eberlein-König B., Schäfer T., Huss-Marp J., Darsow U., Mohrenschlager M., Herbert O., Abeck D., Kramer U., Behrendt H., Ring J. (2000). Skin Surface pH, Stratum Corneum Hydration, Trans-Epidermal Water Loss and Skin Roughness Related to Atopic Eczema and Skin Dryness in a Population of Primary School Children: Clinical Report. Acta Derm. Venereol..

[24] Edwards C., Heggie R., Marks R. (2003). A Study of Differences in Surface Roughness Between Sun-Exposed and Unexposed Skin with Age. Photodermatol. Photoimmunol. Photomed..

[25] Kampf G., Ennen J. (2006). Regular Use of a Hand Cream Can Attenuate Skin Dryness and Roughness Caused by Frequent Hand Washing. BMC Dermatol..

[26] Song J., Vorburger T.V. (1996). Stylus Flight in Surface Profiling. ASME J. Manuf. Sci. Eng..

[27] Tian Y., Liu X., Zhang D., Chetwynd D.G. (2009). Dynamic Modeling of the Fidelity of Random Surface Measurement by the Stylus Method. Wear.

[28] Tian Y., Liu X., Chetwynd D.G., Shirinzadeh B., Zhang D. (2010). Vibration Analysis of Stylus Instrument for Random Surface Measurement. Precis. Eng..

[29] Arvinth Davinci M., Parthasarathi N.L., Borah U., Shaju K.A. (2014). Effect of the Tracing Speed and Span on Roughness Parameters Determined by Stylus Type Equipment. Measurement.

[30] Pawlus P., Smieszek M. (2005). The Influence of Stylus Flight on Change of Surface Topography Parameters. Precis. Eng..

[31] Pawlus P., Reizer R. (2024). Influence of the Traverse Speed of the Stylus Tip on Changes in the Areal Texture Parameters of Machined Surfaces. Materials.

[32] Miller T., Adamczak S., Swiderski J., Wieczorowski M., Letocha A., Gapinski B. (2017). Influence of Temperature Gradient on Surface Texture Measurement with the Use of Profilometry. Bull. Pol. Acad. Sci. Tech. Sci..

[33] Grochalski K., Wieczorowski M., Jakubek B. (2022). Influence of Thermal Disturbances on Profilometric Measurements of Surface Asperities. Measurement.

[34] Nieslony P., Krolczyk G.M., Zak K., Maruda R.W., Legutko S. (2017). Comparative Assessment of the Mechanical and Electromagnetic Surfaces of Explosively Clad Ti–Steel Plates After Drilling Process. Precis. Eng..

[35] Stout K.J., Sullivan P.J., Dong W.P., Mainsah E., Luo N., Mathia T.G., Zahouani H. (1993). The Development of Methods for the Characterisation of Roughness in Three Dimensions.

[36] Hillman W., Kranz O., Eckolt K. (1984). Reliability of Roughness Measurements Using Contact Stylus Instruments with Particular Reference to Results of Recent Research at the Physikalisch-Technische Bundesanstalt. Wear.

[37] Whitehouse D.J. (1974). Theoretical Analysis of Stylus Integration. CIRP Ann..

[38] Pawlus P. (2004). Mechanical Filtration of Surface Profiles. Measurement.

[39] Zahouani H., Vargiolu R., Kapsa P., Loubet J.L., Mathia T.G. (1998). Effect of Lateral Resolution on Topographical Images and Three-Dimensional Functional Parameters. Wear.

[40] Lee D.H. (2013). 3-Dimensional Profile Distortion Measured by Stylus Type Surface Profilometer. Measurement.

[41] Lee D.H., Cho N.G. (2012). Assessment of Surface Profile Data Acquired by a Stylus Profilometer. Meas. Sci. Technol..

[42] Chetwynd D.G. (1979). The Digitization of Surface Profiles. Wear.

[43] Pawlus P., Chetwynd D.G. (1996). Efficient Characterization of Surface Topography in Cylinder Bores. Precis. Eng..

[44] Rosén B.G., Garnier J. (2011). Uncertainties and Optimized Sampling in Surface Roughness Characterization. Wear.

[45] Thomas T.R., Rosen B.G. (2000). Determination of the Optimum Sampling Interval for Rough Contact Mechanics. Tribol. Int..

[46] Pawlus P., Zelasko W. (2012). The Importance of Sampling Interval for Rough Contact Mechanics. Wear.

[47] Pawlus P., Reizer R., Wieczorowski M. (2020). Characterization of the Shape of Height Distribution of Two-Process Profile. Measurement.

[48] Pawlus P., Reizer R., Wieczorowski M. (2021). Functional Importance of Surface Texture Parameters. Materials.

[49] Blateyron F., Leach R. (2014). The areal feature parameters. Characterisation of Areal Surface Texture.

[50] Lagarde J.M., Rouvrais C., Black D. (2005). Topography and Anisotropy of the Skin Surface with Ageing. Skin Res. Technol..

[51] Masuda Y., Oguri M., Morinaga T., Hirao T. (2013). Three-Dimensional Morphological Characterization of the Skin Surface Micro-Topography Using a Skin Replica and Changes with Age. Skin Res. Technol..

[52] Wu Y., Tanaka T. (2021). Objective and Quantitative Measurement of Skin Micro-Relief by Image Analysis and Application in Age-Dependent Changes. Skin Res. Technol..

[53] Cinotti E., Bovi C., Tonini G., Labeille B., Heusèle C., Nizard C., Schnebert S., Aubailly S., Barthélémy J.C., Cambazard F. (2020). Structural Skin Changes in Elderly People Investigated by Reflectance Confocal Microscopy. J. Eur. Acad. Dermatol. Venereol..

